# Student mental health: Beyond the data, questions still need to be asked

**DOI:** 10.1192/j.eurpsy.2023.75

**Published:** 2023-07-19

**Authors:** Y. Morvan

**Affiliations:** ^1^Psychologie, SPSE, CLIPSYD, Université Paris Nanterre, Nanterre; ^2^Psychiatrie du Développement et Trajectoires, Inserm U1018, CESP, Paris, France

## Abstract

**Abstract: Introduction:**

Student mental health was a public health problem long before the pandemic. However, first one needs to define what mental health means. Fried *(2017*) showed that there was a great heterogeneity in the symptoms assessed by the depression scales. Similarly, other factors (study design, assessment period, chosen scale and chosen cut-off or threshold, response rate, data management, etc.) can have an impact on the prevalence found. In addition, theoretical and modelling considerations on mental health need to be answered.

**Methods:**

The french *Observatoire de la Vie Etudiante* measured an increase in student psychological distress since 2016, particularly between 2020 and 2021. Data from three surveys conducted in 2016 (n=18,875), 2020 (n=60,014), and 2021 (n=4,901, longitudinal follow-up from 2020) were used to model psychological distress as a latent common cause or a network with emergent properties.

**Results:**

Preliminary results show that from a latent perspective, measurement invariance does not hold. From a network perspective, the modelled systems showed differences in three aspects (*van Borkulo et al., 2022*). For participants in the 2020 and 2021 surveys, an increased vulnerability of the modelled system was observed. Prevention and intervention targets in the system were tested with simulation techniques (*Lunansky et al., 2022*).

**Conclusion:**

Caution is advised for prevalence comparisons when measurement invariance does not hold. The network approach offers an alternative to studying psychological distress as an emergent property of a complex system. However, regardless of the statistical approach, with subjective measures and without measurement error control and qualitative data or cognitive interviews: it is difficult to partition between a change or increase in the phenomenon we wish to measure and a change in the way people tend to respond to a questionnaire, since the representation they have of the specific questions designed to describe this phenomenon might also have changed.

**Disclosure of Interest:**

None Declared

